# Validation of Analytical Models for the Development of Non-Invasive Glucose Measurement Devices

**DOI:** 10.3390/bios15100669

**Published:** 2025-10-03

**Authors:** Bruna Gabriela Pedro, Fernanda Maltauro de Cordova, Yana Picinin Sandri Lissarassa, Fabricio Noveletto, Pedro Bertemes-Filho

**Affiliations:** 1 Department of Electrical Engineering, Universidade do Estado de Santa Catarina, Joinville 89219-710, Brazil; bruna.pedro@edu.udesc.br (B.G.P.); fabricio.noveletto@udesc.br (F.N.); 2 Centro Universitário Católica de Santa Catarina, Joinville 89203-005, Brazil; fernanda.cordova@catolicasc.org.br (F.M.d.C.); yana.lissarassa@catolicasc.org.br (Y.P.S.L.)

**Keywords:** glucose, molar extinction coefficient, diabetes, spectrophotometry, biosensor

## Abstract

Non-invasive glucose monitoring remains a persistent challenge in the scientific literature due to the complexity of biological samples and the limitations of traditional optical methods. Although advances have been made in the use of near-infrared (NIR) spectrophotometry, the direct application of the Lambert–Beer Law (LBL) to such systems has proven problematic, particularly due to the non-linear behavior observed in complex organic solutions. In this context, the objective of this work is to propose and validate a methodology for the determination of the extinction coefficient of glucose in blood, taking into account the limitations of the LBL and the specificities of molecular interactions. The method was optimized through an iterative process to provide consistent results over multiple replicates. Whole blood and plasma samples from two individuals were analyzed using spectrophotometry in the 700 nm to 1400 nm. The results showed that glucose has a high spectral sensitivity close to 975 nm.The extinction coefficients obtained for glucose ($\alpha_{g}$) ranged from −0.0045 to −0.0053, and for insulin ($\alpha_{i}$) from 0.000075 to 0.000078, with small inter-individual variations, indicating strong stability of these parameters. The non-linear behaviour observed in the relationship between absorbance, glucose and insulin concentrations might be explained by the changes imposed by both *s* and *p* orbitals of organic molecules. In order to make the LBL valid in this context, the extinction coefficients must be functions of the analyte concentrations, and the insulin concentration must also be a function of glucose. A regression model was found which allows to differentiate glucose from insulin concentration, by considering the cuvette thickness and sample absorbance at 965, 975, and 985 nm. It can also be concluded from experiments that wavelength of approximately 975 nm is more suitable for blood glucose calculation by using photometry. The final spectra are consistent with those reported in mid-infrared validation studies, suggesting that the proposed model encompasses the key aspects of glucose behavior in biological media.

## 1. Introduction

For the implementation and maintenance of appropriate treatment for diabetic patients, blood glucose levels become the most important metric. Blood sugar control is essential for both daily management and for preventing harmful long-term effects such as kidney, nerve, eye, and cardiovascular problems [1].

The conventional and most well-known method for continuous glucose monitoring (CGM) is a glucose sensor based on electrochemical and enzymatic reactions. Although accessible, traditional CGM methods still require small blood samples, usually obtained from the fingertip or earlobe. Therefore, the method becomes inconvenient, painful, and difficult to adhere to in certain populations, such as children and the elderly [2].

The development of non-invasive, accurate and precise CGM devices has become a public health urgency in the current landscape of diabetes. Devices based on techniques that eliminate the need for blood sampling offer better quality of life and greater adherence to disease management for patients [3]. One example is the radio wave emission sensor operating at frequencies ranging from 5 to 12 GHz, but it uses high frequencies to minimize the influence of the epithelial barrier and to increase the device’s accuracy [4].

New non-invasive techniques are getting popular in this field of research, such as electrical bioimpedance spectroscopy (EBIS). EBIS has been effectively applied for over 10 years in the characterization of tissues and organic molecules. The technique is based on injecting an alternate current in a wide frequency range in the skin, measures the resulting voltage and then calculated the impedance spectra for extracting the electrical properties of the underlayed structure of the skin, including blood veins [5]. The studies presented by [4,6] proposed low-cost and reliable impedance-based measurement systems capable of correlate a good relationship between glucose concentration variations and skin impedance spectra.

Optical sensors are also good alternatives for exploring the non-invasive blood glucose measuruments [7], herein referred to as spectrophotometry. They use sensors in the range of mid-infrared and near-infrared by exploring the interaction characteristics of photons with the medium, such as scattering, transmission, and light absorption. Spectrophotometry has been widely studied as a technique for measuring blood glucose, especially in the development of non-invasive glucometers. However, its application faces significant challenges. The Lambert–Beer Law, which is fundamental for quantifying analytes in solution, cannot be applied directly to blood due to its complexity as a heterogeneous medium, which contains cells, proteins, and other components that affect optical absorption.

In addition, the absorption of glucose in the near-infrared (NIR) region is relatively weak and overlapped by the absorption of other biomolecules, such as water and plasma proteins. These limitations require sophisticated mathematical models to extract reliable information, increasing the complexity of signal processing. Another challenge is inter-individual variability, as factors such as hematocrit and hydration influence the spectral response.

Recent works have proposed several approaches to address these challenges. For instance, Ghozzi et al. [8] compared linear and non-linear regression models for non-invasive glucose prediction, showing that non-linear models significantly improved estimation accuracy—which supports our approach of revising the classical Lambert–Beer model. Similarly, Choi et al. [9] reviewed advancements in wearable biosensors and emphasized the importance of material and mechanical design in enhancing signal stability—a key factor for implementing analytical models in real-world systems. Zhang et al. [10] provided a comprehensive overview of non-invasive techniques, including optical and hybrid strategies, reinforcing the scientific momentum toward accurate and non-invasive CGMs. Finally, Klyve et al. [11] validated a novel sensor against a Dexcom G6®, highlighting the necessity of experimental validation for analytical models, a goal shared in this present work.

Despite these difficulties, advances in spectral correction techniques, artificial intelligence, and multivariate analysis have improved the accuracy of spectrophotometry, making it a promising alternative for non-invasive glucose measurement, provided its limitations are carefully addressed [12].

**Figure 1:**
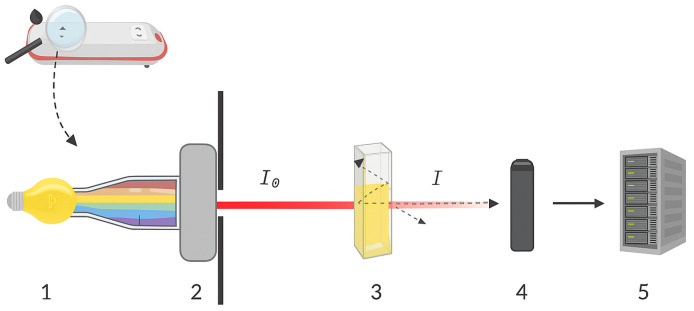
Schematic representation of a photometric instrument. 1 represents the electrical energy source, while 2 represents the monochromator filter. The cuvette with reflection, dispersion and absorption effects is represented by 3. The light detection system and the sensing circuit are represented by 4 and 5, respectively. $I_{0}$ is the incident energy and *I* is the emergent energy.

A spectrophotometer is generally composed of an electrical energy source, a radiant energy source, a monochromator, cuvettes, detectors, a measuring circuit, and a microprocessor. Figure 1 illustrates the basic components of the photometry system.

To meet different analytical demands, various light sources are employed in spectrophotometers. Tungsten lamps are the most commonly used light source for analyses covering wavelengths in the infrared and visible regions of the spectrum, whereas deuterium vapor lamps are employed for analyses in the ultraviolet region [13].

Although the cuvette is a simple component, it represents one of the main sources of error in photometric analyses. Compared with other components of the spectrophotometric system, the cuvette is the item that comes into direct contact with the operator, which implies a higher risk of handling-related errors, including improper manipulation and inadequate cleaning during experimentation.

The material and the design of the cuvette may introduce distinct analytical variations. Quartz cuvettes, with rectangular geometry and polished faces, are particularly suitable for analyses in the infrared region, as they minimize refraction errors and reduce material interference within the working spectral range [13,14].

Beyond cuvette-related issues, spectrophotometers also present inherent technical and analytical limitations. In complex matrices such as human blood, lack of specificity becomes critical, as multiple molecules may absorb at the same wavelength, affecting absorbance and transmittance measurements. Thus, a precise understanding of the optical behavior of the target molecule is essential for selecting an optimal wavelength and minimizing interference from other analytes.

Although non-invasive devices are highly demanded in the market of diabetes control technologies, the calibration processe is very difficult due to a methodological limitation [15]. Pedro and Bertemes-Filho, 2021, proposed a 3-LED pulse glucometer using a mathematical model based on Lambert–Beer’s law and plethysmography [16], resulting in the detection of small volumetric changes in the microvascular tissue under study. Based on a multiparametric device that employs both, optical and electrical techniques, it was shown that this hybrid technique can eliminate the need of constant calibration for such devices.

The need for improvements of existing devices, as well as to develop new ones, should be aligned with overcoming technical and methodological limitations of current applications. Understanding the structural characteristics of glucose and its interaction with the surrounding medium is essential for constructing reliable, reproducible, and accurate measurement systems. In addition, the challenges associated with the clinical approach of traditional CGMs and the methodological obstacles related to existing non-invasive devices open opportunities for clinical and laboratory testing.

The objective of this article is to investigate the molecular optical pattern and behavior of serum glucose through spectrophotometry, focusing on its absorbance profile across a spectrum of wavelengths. To this end, the absorbance behavior of human plasma was analyzed as a function of glucose and insulin concentrations. Furthermore, this study aims to validate the molar extinction coefficient proposed in our previous work [17] in order to establish an equation for estimating glucose levels based on the extinction coefficient.

## 2. Materials and Methods

The present study was designed and conducted based on fundamental theoretical and practical principles. Drawing from existing literature and previous research, a set of guiding equations was employed, which are presented in the following section. These foundational equations were applied and adapted according to the specific objectives and analytes investigated in this study.

To ensure the practical feasibility of the work, the project was submitted for approval by a research ethics committee. The experimental design incorporated participant inclusion and exclusion criteria to ensure both safety and population appropriateness. Laboratory measurements of the analytes (glucose and insulin) were performed using gold-standard methodologies to maximize methodological precision and accuracy, thereby enhancing the reliability and validity of the data obtained.

Data processing and analysis were conducted by following established protocols and guided equations, which was adaptated to the aims of this study.

### 2.1. Governing Equations

When a light beam (visible or not) passes through a solution, the intensity of the incident energy ($I_{0}$) will always be greater than the emergent energy (*I*). Reflection at the air-cuvette interface, dispersion of particles, and energy absorption by analytes are responsible for this attenuation of $I_{0}$ [13].

In both absorption and scattering processes (reflection and dispersion), the incident light intensity $I_{0}$ decreases as it passes through the medium, resulting in *I*, as seen in Figure 1. This attenuation is called light extinction. The extinction coefficient α is defined as a combination of the absorption coefficient $\alpha_{abs}$ and the scattering coefficient $\alpha_{scat}$ for all particles in the medium [14]. Mathematically:

(1) $$\alpha =\alpha_{abs}+\alpha_{scat}$$

The molar extinction coefficient, also referred to as molar absorptivity, absorption coefficient, or molar absorptivity coefficient, represents the extent to which a mole of a substance attenuates light at a given wavelength [18]. Each chemical species has a specific molar absorptivity value for a given wavelength. The usual unit for α is ${\mathrm{L.mol}}^{-1}{\mathrm{.cm}}^{-1}$ [19].

Light absorption is the primary factor in energy reduction in photometry applications. According to the Lambert–Beer Law (LBL) [14,20], this can be expressed as:

(2) $$I=I_{0}e^{-\alpha lc}$$

where *l* is the optical path length in the medium, and *c* is the analyte concentration.

The fraction of transmitted to incident energy defines the solution’s transmittance *T* [21]:

(3) $$T=\frac{I}{I_{0}}$$

In the absence of absorbance, *I* = $I_{0}$ and *T* = 1. Any absorbing solution will have a transmittance lower than 1.

Rearranging Equation (2) yields:

(4) $$-log\left(\frac{I}{I_{0}}\right)=\alpha lc=A$$

where *A* is the absorbance.

When multiple active substances absorb at the same wavelength, absorbance is described as [22]:

(5) $$A=(\alpha_{1}c_{1}+\alpha_{2}c_{2}+\ldots +\alpha_{n}c_{n})l$$

where $c_{x}$ and $\alpha_{x}$ are the concentration and extinction coefficient of each substance, with x=1,2,…,n. These values can be determined by solving a system of *n* equations with *n* variables.

If n→∞, the sum becomes an infinite weighted series, whose nature depends on the behavior of the coefficients $\alpha_{x}$, $c_{x}$, and the convergence of the series $\sum_{x=1}^{\infty }\alpha_{x}c_{x}$. Since infinite series cannot be handled practically, truncation is necessary. The truncation point depends on the acceptable error margin.

For this research, truncation was chosen at x=2, where the only contributions are from glucose and insulin:

(6) $$A=(\alpha_{g}c_{g}+\alpha_{i}c_{i})l$$

### 2.2. Sample Collection and Handling

This study was submitted for review by the ethics committee for research involving human subjects and was approved under number 6.318.291. The sample consisted of two study participants, one man and one woman, both healthy and meeting the inclusion criteria established in the methodological design.

Healthy men and women aged between 18 and 50 years met the inclusion criteria. Candidates who did not complete the screening and eligibility process for the test were automatically excluded. The diagnosis of autoimmune conditions and non-communicable chronic diseases, such as hypertension, asthma, and diabetes, constituted exclusion criteria for the study. Pregnant candidates, regardless of gestational age, were also not allowed to participate.

In addition, eligible participants with symptoms of fever or general malaise at the time of the experimental procedure were withdraawn from the study due to individual safety reasons.

However, participants who failed to comply with the required fasting period (8 to 12 h) were also considered an exclusion criterion. All criteria were established to ensure the safety of the study population.

Each subject underwent venous blood collection, performed by a trained phlebotomist following the guidelines and recommendations of the Ministry of Health [23] and the Brazilian Society of Clinical Pathology and Laboratory Medicine [24].

Four whole blood samples were collected from each subject, with the number of samples determined based on the technical adaptation of the oral glucose tolerance test (OGTT) to the study objectives. In this case, one sample was collected while fasting, and the others were taken at 30-min intervals after a 75 g glucose load, following the initial fasting sample. Whole blood samples were obtained using a closed venous blood collection system (vacuum puncture) and were directly collected into fluoride-containing tubes.

It should be emphasized that sodium fluoride, in addition to acting as an anticoagulant, inhibits the enzyme enolase, thereby blocking glycolytic pathway progression. This action prevents glucose from being consumed as an energy source by blood cells, ensuring that glucose concentration is not reduced due to physiological activity, thus, providing greater accuracy and reliability for glucose measurement.

The samples were gently homogenized to mix the blood with the fluoride and were centrifuged 30 min after collection. Adhering to the interval between collection and centrifugation is critical to guarantee the anticoagulant effect and the inhibition of the glycolytic pathway by fluoride.

Plasma samples were analyzed using a automatic commercial biochemical device for glucose measurement (mg/dL), properly calibrated and validated with high- and low-level control sera. The methodology used was enzymatic colorimetry based on spectrophotometry. Insulin levels were determined using chemiluminescence.

**Figure 2:**
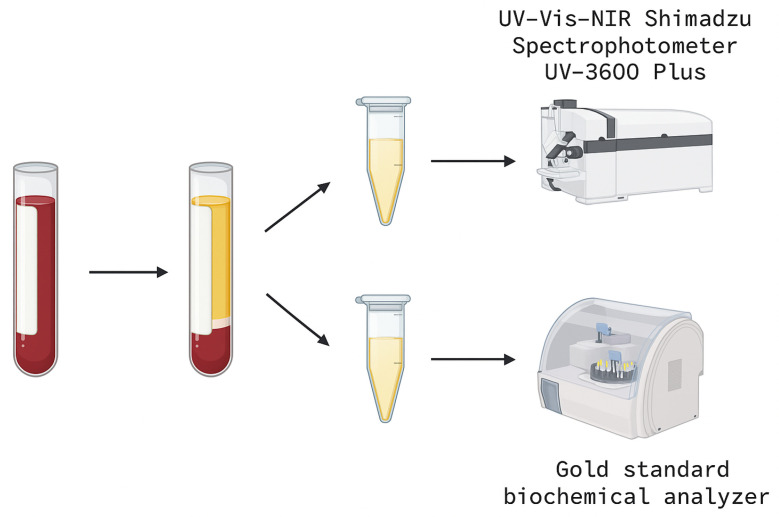
Pre-analytical and analytical flow of the collected samples.

Figure 2 represents the sample separation process. The samples were centrifuged to obtain fluoridated plasma and subsequently aliquoted. The aliquots were sent for distinct quantitative analyses.

The total time between sample collection and both the biochemical and optical analyses did not exceed 3 h. However, between collection, processing, screening, and analysis, the aliquots were stored in appropriate containers and at controlled temperatures to ensure the physiological stability of the samples.

### 2.3. Sample Processing and Analysis

Plasma samples assigned to optical analysis were analyzed using the UV–Vis–NIR spectrophotometer (model UV3600Plus) from Shimadzu (Barueri, Brazil), with a wavelength scan ranging from 700 nm to 1400 nm to study the absorbance pattern behavior. Quartz cuvettes of 1 mm were used as the optical path. Water was used as the photometric blank.

The selected wavelength range accounted for known plasma interferents, such as albumin and various globulins—the most abundant proteins in plasma. It is well established that albumin has greater reactivity at 620 nm, while globulins react strongly in the range of 430 nm to 450 nm, as evidenced by [25,26], respectively.

One of the most significant colorimetric interferents in blood spectrophotometry is hemoglobin, which exhibits higher absorptivity at wavelengths of 540 nm and 580 nm [27]. In this case, pigment interference is disregarded due to the exclusive use of plasma. However, the standard protocol’s chosen wavelength range could also mitigate hemoglobin interference in whole blood analysis.

Using this range neither excludes other absorbing molecules responding to the same wavelengths nor rules out their relevance in glucose’s optical behavior. This gap allows future studies on the photometric behavior of other plasma components present in significant amounts during the glucose peak regulation cycle.

### 2.4. Absorbance Measurements and Extinction Coefficient Calculation

The results presented in this article are part of an iteractive process. Previous experiments confirmed the repeatability of results [28] regarding blood absorption for different glucose concentrations. However, methodological refinement was necessary to calculate the extinction coefficient in blood.

Over the course of one year of experimentation, three distinct experimental procedures were conducted by varying the spectrophotometer, wavelength range, analyte, subjects, and the cuvette thickness. The resume is presented in Table 1.

**Figure 3:**
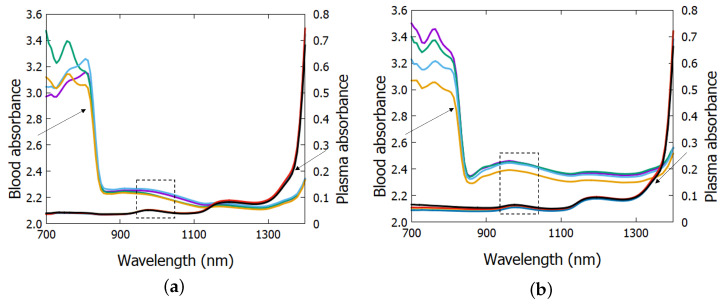
Absorbance spectrum for wavelengths from 700 nm to 1400 nm of plasma and blood, where (**a**) represents data from subject 1 and (**b**) from subject 2.

In the first experiment, significant interference was observed, initially attributed to hemoglobin presence. In the second experiment, the cuvette thickness was reduced, decreasing the mean free path (*l*) in Equation (6). Spectrophotometry graphs of whole blood and plasma (Figure 3) revealed that absorbance values for wavelengths (λ) below 900 nm in whole blood were unusable. The figure also shows an absorption peak at 975 nm for both samples (blood and plasma) in both subjects, indicating a significant absorption range for blood glucose analysis. It is important to emphasize that the experiment consisted of the ingestion of glucose syrup between fasting and the end of sample collection. This justifies the calculation of the results in the following section using wavelengths of 965, 975, and 985 nm.

Another reason for this choice is based on the assumption that *l* is equal for all measurements in Equation (6), which is valid only for closely spaced wavelengths. When wavelengths are close, the optical medium can be considered “homogeneous” with respect to its light response, as absorption and scattering mechanisms remain similar within this narrow range. For more distant wavelengths, the medium may interact differently with light, significantly altering the mean free path [17,29].

The results of [28], particularly the absorbance graphs as a function of glucose concentration, highlight the nonlinearity with wavelength. This behavior motivated the inclusion of insulin dosage as a differentiator in this last experiment. During glucose measurements, it was known that other blood components underwent changes in their volume fractions. However, their contributions to absorption were mitigated by using fasting samples as blanks. Nevertheless, insulin was considered the most significant analyte that could not be ignored in improving the methodology.

In summary, this study employs curve regression of Equation (6) using data on glucose concentration, insulin concentration, cuvette thickness, and sample absorbance at 965, 975, and 985 nm.

## 3. Results

The Table 2 presents the results obtained using the methodology described in the previous section. These results reflect the final stage of experimentation, which involved both male and female subjects and included insulin analysis. Glucose measurements were converted to mmol/L due to dimensional analysis, while insuline is given in uUI/mL. *l* was expressed in cm in Equation (6).

Glycemic and insulinemic variations can be observed among the participants. The regulation of glycemic peaks does not rely solely on innate variables, such as pancreatic insulin production, sex, or age. Sleep quality, physical activity, diet, fluid intake, and body composition are also fundamental factors in glycemic regulation [30].

Despite the significant variations in glucose and insulin levels observed among participants, the analysis of the absorbance profiles revealed a highly similar optical behavior between the two. This finding may suggest that human plasma from healthy individuals exhibits a standardized optical response, which could serve as a reference optical spectrum.

According to the linearity proposed by the Lambert–Beer Law, solutions with higher solute concentrations are expected to exhibit greater absorbance. However, this trend is not observed within the wavelength range presented in Table 2.

For instance, sample T60 from participant 1 contains 2.44 mmol/L more glucose and 114.25 µIU/mL more insulin compared to sample T30. Nevertheless, its absorbance is either equal (=975 nm) or even lower (=965–985 nm) than that of the sample with lower solute concentrations.

The data obtained from participant 2 indicates that the peak concentrations of both glucose and insulin occurred at T30, whereas the highest absorbance values were at T60. Based on the corresponding biochemical and optical analyses, it is not possible to infer, within this wavelength range, a more significant optical predominance of either glucose or insulin, since the concentration and absorbance peaks are not concordant.

**Figure 4:**
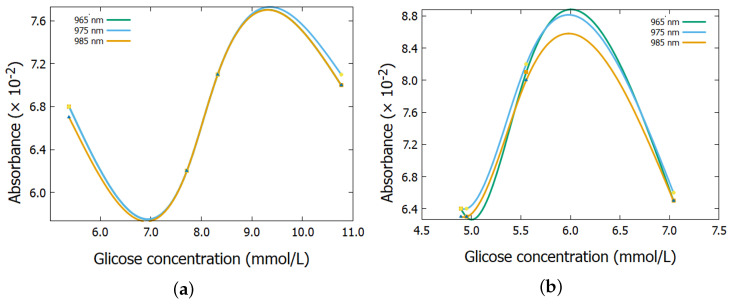
Absorbance spectrum as a function of glucose concentration for (**a**) subject 1 and (**b**) subject 2.

**Figure 5:**
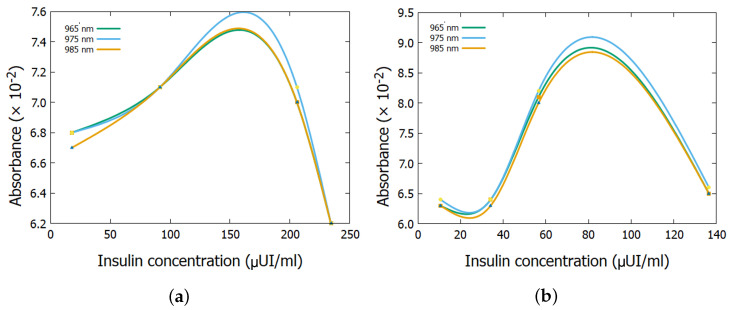
Absorbance spectrum as a function of insulin concentration for (**a**) subject 1 and (**b**) subject 2.

Figure 4 and Figure 5 show the absorbance spectrum as a function of glucose concentration for the two subjects, considering the wavelengths of 965 nm, 975 nm, and 985 nm. It is observed that, in both cases, the curves exhibit a nonlinear behavior, similar to previous tests [28], thus, reinforcing the deviation from the linear pattern predicted by the Lambert–Beer Law, as previously observed in the analysis of the results presented in Table 2.

In Table 3, the curve regression parameters obtained using GNUplot are organized for the wavelengths of 965, 975, and 985 nm. Here, $\alpha_{g}$ represents the glucose extinction coefficient, and $\alpha_{i}$ represents the insulin extinction coefficient.

For $\alpha_{g}$, the differences between wavelengths are on the order of $10^{-4}$ or $10^{-5}$, while the absolute value is on the order of $10^{-3}$. This relative variation is very small, especially considering experimental precision. For $\alpha_{i}$, the variation is even smaller, on the order of $10^{-6}$, reinforcing the idea that the parameter is stable.

The values of $\alpha_{g}$ and $\alpha_{f}$ do not follow a clear trend that systematically relates them to wavelength, suggesting that their small fluctuations may be attributed to experimental noise or minor metabolic differences between subjects.

Both subjects exhibit very similar values for $\alpha_{g}$ and $\alpha_{i}$, further indicating that these parameters can be considered constant or nearly constant within the analyzed range.

The regression models demonstrated high visual agreement between experimental absorbance data and fitted curves. However, it needs to be emphasized that the coefficients of determination (R^2^) were not computed here, which may lead to a limitation in this study. Future validation will incorporate such indicators to quantitatively assess model performance.

## 4. Discussions

Figure 4 and Figure 5 present nonlinear behaviors that can be explained through nonlinear optics, which deals with the behavior of light in nonlinear media. In these media, the dielectric polarization *P* of the material is not proportional to the electric field *E* of the light [31,32,33]. Nonlinear effects occur at high light intensities, such as laser radiation. The mathematical representation of polarization, under simple circumstances, can be expressed as:

(7) $$\tilde{P}\left(t\right)=\varepsilon_{0}\left[\chi^{\left(1\right)}\tilde{E}\left(t\right)+\chi^{\left(2\right)}{\tilde{E}}^{2}\left(t\right)+\chi^{\left(3\right)}{\tilde{E}}^{3}\left(t\right)+\ldots \right]$$

where $\chi^{\left(1\right)}$ is the linear electric susceptibility tensor, $\chi^{\left(2\right)}$ is the second-order susceptibility, and $\chi^{\left(3\right)}$ is the third-order susceptibility, among others. $\tilde{P}$ and $\tilde{E}$ represent tensorial entities of *P* and *E* [33].

The second term in Equation (7), with $\chi^{\left(2\right)}$ present in non-centrosymmetric materials, is responsible for second-order nonlinear optical effects such as second harmonic generation, sum-frequency generation, difference-frequency generation, and the linear electro-optic effect (Pockels effect). The third term, with $\chi^{\left(3\right)}$, accounts for third-order nonlinear optical effects, including third harmonic generation, four-wave mixing, Raman and Brillouin scattering, self-phase modulation, cross-phase modulation, and the quadratic electro-optic effect (Kerr effect). This term exists in media with or without inversion symmetry [31,32,33].

The *Kerr effect* describes a change in the refractive index (Equation (8)) induced by the light beam itself. When intense, the beam can act as its own modulating electric field without requiring an externally applied electric field [32]. The refractive index *n* depends on the linear part $n_{0}={{(}^{1}+\chi^{\left(1\right)})}^{\frac{1}{2}}$ and the nonlinear part ${\tilde{n}}_{2}=\frac{3}{8}\frac{\chi^{\left(3\right)}}{n_{0}}$ [31].

(8) $$n_{0}+{\tilde{n}}_{2}{\left|E_{0}\right|}^{2}$$

Assuming a significant variation in the medium’s refractive index due to changes associated with glucose metabolism (e.g., insulin intake, variations in water and electrolyte content in the sample volume), a reverse analogy to the Kerr effect can be explored. In this case, it is not the laser that modifies the medium’s refractive index, but rather the refractive index variation itself that alters the transmitted light intensity nonlinearly, thereby affecting the absorbance calculation.

On the other hand, studies on nonlinear optics in organic materials claim that nonlinearity is an inherent characteristic of such samples [34,35,36,37]. Carbon atoms have *s* and *p* atomic orbitals and are generally the main components of organic molecules. When bonded, they form molecular orbitals σ and π. The out-of-plane π orbital is strongly affected by the electric field of the incident laser radiation, causing distortions that result in nonlinear optical phenomena [34,37].

In this context, the occurrence of negative extinction coefficients in our experimental data can be understood as a manifestation of such nonlinearities. A negative coefficient may be interpreted either as an effective optical gain, as described in active media with stimulated emission [38], or as a consequence of the complex refractive index formalism, where the imaginary part becomes negative [33,39]. Both perspectives are consistent with the nonlinear optical response induced by the π-bonds of glucose and related hydrocarbon structures under high-intensity illumination.

The consequences of these results need reassessment of mathematical models based on the Lambert–Beer law, as described in Equation (2), as presented in [17,40]. The Lambert–Beer law is strictly valid only under ideal conditions. In practice, several phenomena can cause deviations from linearity, such as high concentrations, scattering effects, fluorescence, or other photochemical reactions.

The lack of linearity in the graphs may indicate that the coefficients $\alpha_{x}$ are not constant across all concentrations, but this does not invalidate the model itself. If $\alpha_{k}$ varies with concentration, $\alpha_{x}=f_{x}\left(c_{x}^{2}\right)$, the model remains valid but with an extinction coefficient that depends on concentration. Furthermore, if glucose and insulin concentrations are interdependent, i.e., $c_{i}\left(c_{g}\right)$, then the system is no longer independent, introducing higher-order terms. Equation (6) still represents the sum of contributions, but these contributions are no longer strictly linear. Accordingly, the model can be reformulated as:

(9) $$A=\left(\alpha_{g}c_{g}+\alpha_{i}c_{i}\right)l=\left(f_{g}\left(c_{g}^{2}\right)c_{g}+f_{i}\left(c_{i}^{2}\right)c_{i}\right)l$$

where $f_{g}\left(c_{g}^{2}\right)=a_{1}c_{g}^{2}+a_{2}c_{g}+a_{3}$ and $f_{i}\left(c_{i}^{2}\right)=b_{1}c_{i}^{2}+b_{2}c_{i}+b_{3}$, with $a_{j}$ and $b_{j}$ (j=1,2,3) being constants. Then:

(10) $$A=\left(a_{1}c_{g}^{3}+b_{1}c_{i}^{3}+a_{2}c_{g}^{2}+b_{2}c_{i}^{2}+a_{3}c_{g}+b_{3}c_{i}\right)l$$

As a result of these equations, the non-linear behaviour of the experiments may justify the results in Figure 4 and Figure 5.

In a previous study [17], the goal was to develop an analytical model free of calibrations. However, the methodology applied with only two analytes does not enable such a result, allowing only the calculation of coefficients for each individual. If the concentration of other analytes (proteins, water, etc.) were known such that Equation (5) had n→∞, the series $\sum_{x=1}^{\infty }\alpha_{x}c_{x}$ would be convergent. This argument is supported by the fact that the differences in extinction coefficients among subjects, shown in Table 2, are small. Therefore, the contribution of additional terms in the series for 3<x<∞ diminishes as x→∞, ensuring series convergence.

Additionally, the absorbance results presented in Figure 3a,b reveal distinct spectral features in the 900–1200 nm region, which match with the glucose absorption patterns identified in clinical studies employing mid-infrared spectroscopy [41]. Although the spectral regions differ, these overlaps reinforce the presence of glucose-related vibrational modes also detectable by NIR-based approaches. This correlation suggests that the analytical models used here are sensitive to biologically relevant variations in glucose concentration, thus, supporting their potential use in non-invasive biosensor development. In line with recent advancements in wearable biosensors [9], our results provide a foundation for future integration of such models into photonic devices aimed at continuous glucose monitoring.

Despite the limited number of subjects included in this study, several factors help mitigate this limitation and support the robustness of the findings. The extinction coefficients for glucose and insulin exhibited minimal variation between the two individuals, indicating high parameter stability across different biological matrices. The experimental protocol was carefully designed and iteratively refined to reduce methodological variability, incorporating validated analytical techniques and strict sample handling procedures.

Additionally, the nonlinear behavior observed in the absorbance curves was theoretically substantiated through nonlinear optics and molecular interaction models, reinforcing the scientific validity of the proposed framework. The selected spectral range (965–985 nm) enabled effective isolation of glucose-related signals, minimizing confounding effects from other biomolecules.

As such, this study provides a solid experimental and analytical foundation for future research with larger cohorts, validating its relevance as a methodological benchmark in the development of non-invasive glucose sensing technologies.

## 5. Conclusions

This work investigated the glucose variations with respect to its absorbance pattern in order to validate the molar extinction coefficient published in our previous work. A good curve regression was found which allows us to differentiate glucose from insulin concentration, taking into account cuvette thickness and sample absorbance at 965, 975, and 985 nm. It can also be concluded from the experiments that a wavelength of approximately 975 nm is more suitable for blood glucose calculation using photometry. In addition, the spectral results demonstrate compatibility with those obtained from mid-infrared validation studies, suggesting that the proposed model captures essential features of glucose behavior in biological media. This supports the clinical relevance of the approach.

Once the extinction coefficients of glucose and insulin are properly determined according to the proposed model here, then other analytical models may take advantage of these findings. The development of non-invasive glucose measurement devices based on photometry may also access the findings of this work in order to reassess the appropriate sensitivity range for glucose detection, since the understanding of the optical dynamics of human plasma paves the way for new analytical modeling approaches.

Future work might benefit from incorporating a broader range of analytes, including proteins and metabolites, to enhance the generalization and robustness of the model. This would further align with the current trend toward integrated, multi-analyte wearable biosensors for continuous and non-invasive health monitoring.

One way to validate the established calculation of the coefficients is by repeating the test with the same volunteers under the same conditions. By obtaining new results and recalculating the coefficients, it will be possible to assess if the coefficients can be consistently established for each individual, thereby suggesting the possibility of individual calibration based on the calculated coefficients.

## Figures and Tables

**Table 1:** Characteristics of the experiments undertaken over the last year according to this methodology.

	Experiment 1	Experiment 2	Experiment 3
Spectrophotometer	Manual scan	Automated scan	Automated scan
Wawelengths	740, 810, 850, 940 nm	700–1400 nm	700–1400 nm
Analytes	Glucose	Glucose	Glucose, Insulin
Subjects	2 Female	2 Female	1 Male, 1 Female
Cuvette thickness	10 mm	1 mm	1 mm

**Table 2:** Glucose and insulin concentrations over time from two subjects, and absorbance values at three different wavelengths.

	Time	Glucose Concentration	Insulin Concentration		Absorbance	
Subject 1	(min)	(mmol/L)	(uUI/mL)	965 nm	975 nm	985 nm
	0	5.38	17.87	0.068	0.068	0.067
	30	8.32	91.55	0.071	0.071	0.071
	60	10.76	205.80	0.070	0.071	0.070
	90	7.71	234.20	0.062	0.062	0.062
Subject 2	(min)	(mmol/L)	(uUI/mL)	965 nm	975 nm	985 nm
	0	4.95	10.66	0.063	0.064	0.063
	30	7.04	136.50	0.065	0.066	0.065
	60	5.55	56.61	0.081	0.082	0.080
	90	4.89	34.16	0.064	0.064	0.063

**Table 3:** Values of the parameters $\alpha_{g}$ and $\alpha_{i}$ obtained from curve regression for two subjects at different wavelengths (965 nm, 975 nm, and 985 nm). The values of $\alpha_{g}$ and $\alpha_{i}$ represent the absorption coefficients for the respective wavelength ranges.

	Extinction Coef.	965 nm	975 nm	985 nm
Subject 1	$\alpha_{g}$	−0.00511378	−0.00526921	−0.00523117
	$\alpha_{i}$	0.00007524	0.00007669	0.00007607
Subject 2	$\alpha_{g}$	−0.00452825	−0.00451808	−0.00474027
	$\alpha_{i}$	0.00007619	0.00007668	0.00007833

## Data Availability

Data are contained within the article.
